# Do anabolic nutritional supplements stimulate human growth hormone secretion in elderly women with heart failure?

**DOI:** 10.14814/phy2.13366

**Published:** 2017-08-04

**Authors:** Ellen T. H. C. Smeets, Scott E. Schutzler, Jeanne Y. Wei, Gohar Azhar, Robert R. Wolfe

**Affiliations:** ^1^ Department of Human Nutrition Wageningen University Wageningen The Netherlands; ^2^ Department of Geriatrics University of Arkansas for Medical Sciences Little Rock Arkansas

**Keywords:** Amino acids, arginine, lysine

## Abstract

Growth hormone treatment has gained attention over the past decade as a treatment for heart failure. Human growth hormone (HGH) must be administered by injections (usually daily), so there is considerable advantage to stimulation of endogenous secretion by amino acid‐based nutritional supplementation. However, studies investigating the effect of amino acid (AA) supplementation show conflicting results. Therefore, in this study we aimed to investigate the effect of nutritional supplementation on HGH production in elderly women with heart failure. Eight elderly women with heart failure participated in this randomized cross‐over study. Plasma HGH concentration was measured before and for 4 h following ingestion of a mixture of protein, carbohydrate, and fat or an AA beverage. HGH concentration was determined with ELISA kits and AA concentrations were analyzed by Liquid Chromatography–Mass Spectrometry (LCMS). Linear mixed models was performed to analyze the effect of time, treatment, and interaction. Plasma arginine and lysine concentrations were significantly higher after consumption of the AA drink compared to the mixture of protein, carbohydrate, and fat. Nonetheless, only ingestion of the protein, carbohydrate, and fat mixture (meal replacement) increased HGH concentration. HGH concentration was increased in elderly women with heart failure following consumption of a meal replacement containing protein, carbohydrate, and fat. Consumption of a mixture of amino acids failed to increase HGH concentration despite significantly greater elevations in plasma amino acid concentrations, including arginine and lysine. The stimulatory effect of the protein/carbohydrate/fat mixture was presumably mediated by factors other than increases in free amino acid concentrations.

## Introduction

Heart failure is the leading cause of death and hospitalization in Western Countries (Lombardi et al. 2014; Carubelli et al. 2015). Not surprisingly, it is estimated that heart failure directly and indirectly is responsible for $30.7 billion of the annual healthcare costs in the United States only and is estimated to even increase to $69.7 billion in 2030 (Mozaffarian et al. 2016). Despite the introduction of new drugs and treatments for patients with heart failure over the past few years, the prevalence of heart failure continues to increase and the quality of life remains poor (Heidenreich et al. 2013; Carubelli et al. 2015).

One treatment for heart failure patients that is gaining attention over the past decade is human growth hormone (HGH). HGH has been shown to correct abnormal vascular reactivity in chronic heart failure patients (Napoli et al. 2002; Andreassen 2010). Moreover, HGH is an important anabolic mediator and therefore has therapeutic potential impact on heart failure patients, since chronic heart failure results in a catabolic state of the body and loss of physical function (Cicoira et al. 2003; Hakuno et al. 2015).

HGH therapy must be administered by injections, usually given daily. Alternatively, HGH levels in the body can potentially be increased by stimulation of endogenous secretion by nutritional supplements, particularly those based on amino acids. The amino acids arginine, methionine, phenylalanine, lysine, and histidine have been shown to promote HGH secretion in adults (Knopf et al. 1965; Chromiak and Antonio 2002). However, studies investigating the effect of amino acid supplementation on HGH levels show conflicting results (Andreassen 2010; de Lemos and Vigen 2014). Greater and more consistent increases in HGH concentrations after amino acid consumption were found in women as compared to men (Chromiak and Antonio 2002). Therefore, in this study we aimed to investigate the effect of nutritional supplementation with a meal replacement beverage (Ensure Active Heart Health) or an amino acid beverage containing essential amino acids plus citrulline and carnitine designed for stimulation of muscle function in heart failure (AA drink) on HGH production in elderly women with heart failure.

## Methods

Written Informed Consent was obtained from each of the subjects participating in the study. The studies were conducted under the approval of the University of Arkansas Institutional Review Board and procedures were performed in accordance with all ethical standards of the review committee and with the Helsinki Declaration of 1975, as revised in 2008.

### Subjects

Eight elderly (80 ± 7 years) women with heart failure participated in this randomized cross‐over study. Heart failure was defined as New York Heart Association (NYHA) class I or II. Subjects took at least one of the following classes of medications; (1) angiotensin converting enzyme inhibitor (ACE inhibitor), (2) *β*‐blocker, (3) diuretics, and (4) angiotensin II receptor blockers. Exclusion criteria were anemia (hemoglobin < 10.0g/dL), history of a chronic inflammatory condition or disease, myocardial infarction in the past 6 months, unstable angina, moderate to severe valvular heart disease, significant ventricular arrhythmias, moderately severe dementia (MMSE < 18), history of malignancy in the past 6 months, cancer chemotherapy or radiation within 1 year, HbA1C > 9.0, insulin usage for diabetes control, chronic kidney disease (GRF < 30), more than 5% body weight loss during the past 6 months, or documented infiltrative, restrictive or hypertrophic cardiomyopathy.

### Study visits

The experimental trials were performed in a randomized cross‐over design. Once accepted into the study, subjects took part in two experimental test days. Each test day consisted of a 7‐h infusion period, during which three stable isotopes were infused [ring‐^2^H_5_‐phenylalanine, ^2^H_2_‐tyrosine, and ^2^H_4_‐tyrosine] (results of isotopic data presented elsewhere). The first 3 h of the tracer infusion was in the postabsorptive state following an overnight fast. At 3 h subjects ingested either the AA drink or the meal replacement (Ensure Active Heart Health (Abbott Nutrition)) drink. Blood samples for the analysis of HGH were collected in serum separator collection tubes after 120 (basal), 220 (40 min after ingestion) and 280 (100 min after ingestion) minutes of infusion. Blood samples for the analysis of AA concentrations were collected in EDTA collection tubes at baseline and after 2 h of infusion at 20 min intervals for 5 h.

### Meal replacement drink

A bottle of Ensure Active Heart Health chocolate drink (Abbott Nutrition) of 237 mL consisted of 8 g of protein added in the form of milk protein isolate and soy protein isolate. Additionally, this drink consisted of 3 g of fat and 21 g of carbohydrates. One bottle of chocolate drink contained 410 kilocalories (kCal).

### AA drink

The AA drink was supplied by Prinova Inc, Chicago, IL, and consisted of the following amino acids: histidine, isoleucine, leucine, lysine, methionine, phenylalanine, threonine, valine, tryptophan, citrulline, and L‐carnitine (60 kCal). The AA drink was made by dissolving 15 g of the powdered formulation in 240 mL of distilled water.

### Analysis of HGH

All blood samples were analyzed with both the human HGH ELISA kit with a detection range from 2.5 to 50 ng/mL (ALPCO, Salem, NH) and the Ultrasensitive HGH ELISA kit range 25–1600 pg/mL (R&D Systems, Minneapolis, MN). Lyphochek Immunoassay Plus Controls levels 1, 2, and 3 (BioRAD, Irvine, CA) were used as control samples for the standard curve of the HGH ELISA 2.5–50 ng/mL.

### Analysis of amino acid concentrations

Plasma amino acid concentrations for all blood samples were analyzed using Liquid Chromatography Mass Spectrometry (LC–MS) at the University of Arkansas for Medical Sciences, Center for Translational Research in Aging and Longevity lab.

### Statistical analysis

All data are presented as means and the standard error of the mean. The effect of time, treatment and interaction [time*treatment] on plasma HGH levels and amino acid levels were analyzed with linear mixed models. Bonferroni correction was performed for the pairwise comparison between both treatments. The level of significance was set at *P* < 0.05. All analyses were performed using SPSS 23 (SPSS Inc. Chicago IL).

## Results

### Baseline characteristics

The mean age of the eight elderly women included in our cross‐over study was 80 ± 7 years. Average BMI was 32.7 ± 5.9 kg/m^2^. The average systolic blood pressure was 145 ± 17 mmHg, the mean diastolic blood pressure was 75 ± 5 mmHg and the average heart rate was 73 ± 12 beats/min. Baseline characteristics for all eight women are shown in Table 1.

**Table 1:** Baseline characteristics of all eight elderly women included in this cross‐over study

	Subjects [*n* = 8]
Age [years]	80 ± 7^a^
BMI [kg/m^2^]	32.7 ± 5.9
Current smoker [*n*, %]	0 [0%]
Systolic BP [mmHg]	145 ± 17
Diastolic BP [mmHg]	75 ± 5
Heart rate [beats/min]	73 ± 12
MMSE	28 ± 2
Medication [*n*, %]^b^	
ACE inhibitor	4 [50%]
* β*‐blocker	3 [37.5%]
Diuretics	6 [75%]
Angiotensin II receptor blockers	0 [0%]

MMSE, Mini‐mental state examination.

a Mean ± SD, all such values.

b Some subjects took a combination of medication subclasses.

### Human growth hormone analysis

A significant effect on HGH levels was observed over time (*P* = 0.019). No significant differences between both treatments (meal replacement vs. AA) were found (*P* = 0.476). Additionally, no interaction effect was found (*P* = 0.300; Table 2).

**Table 2:** Results of the linear mixed model for average growth hormone response

	*F* value	*P* value
Time effect	4.436	0.019
Treatment effect	0.518	0.476
Time × treatment interaction	1.245	0.300

### Meal replacement versus AA drink

**Figure 1:**
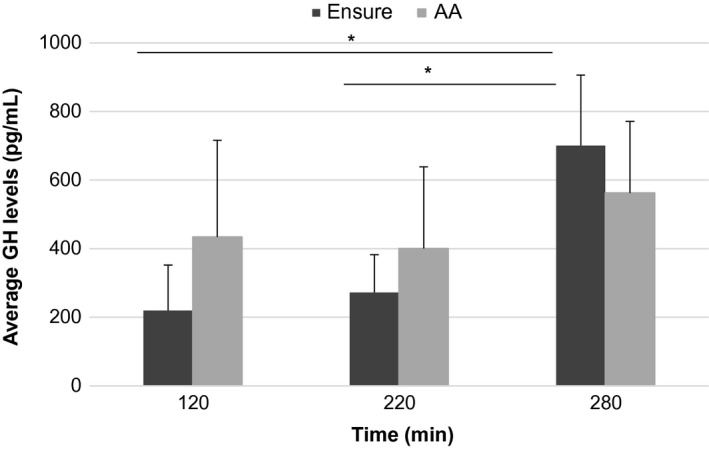
Average plasma growth hormone concentrations after the meal replacement and amino acid (AA) drink at time point 120, 220, and 280. *P < 0.05.

Separate analysis for both treatments revealed significant differences between the basal value and time points 220 and 280 for the meal replacement drink (*P* = 0.018 and *P* = 0.040, respectively). No significant differences between time points were observed for the AA drink (*P* = 1.000 and *P* = 0.994, respectively; Fig. 1).

### Amino acid concentrations

**Figure 2:**
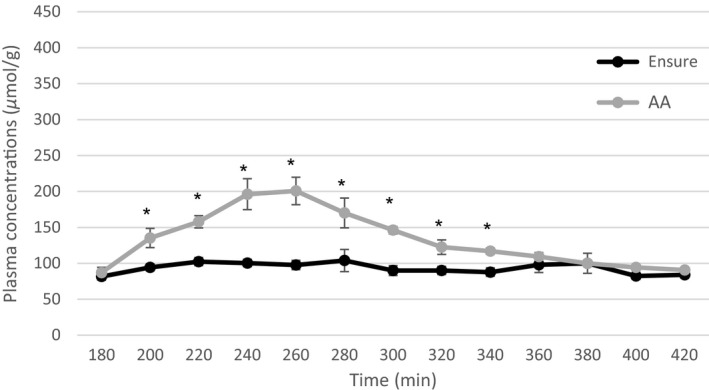
Average plasma arginine levels after the meal replacement and AA drink for time point 180–420 min, *P < 0.05.

**Figure 3:**
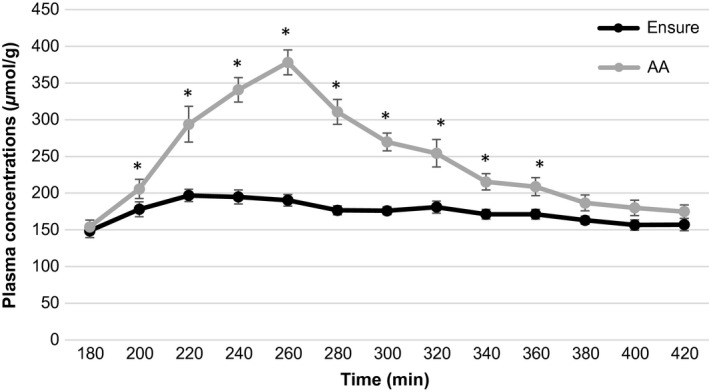
Average plasma lysine levels after the meal replacement and AA drink for time point 180–420 min.

**Figure 4:**
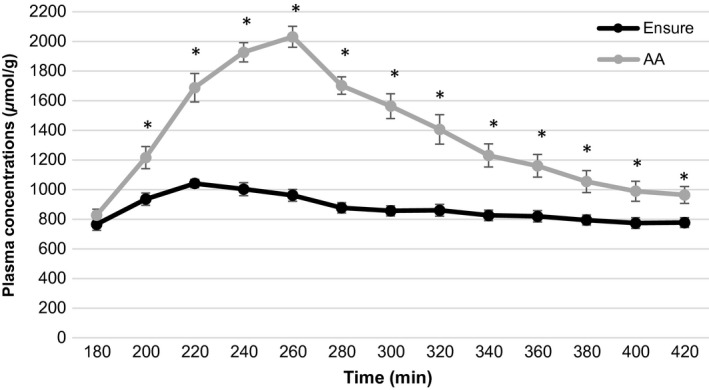
Average plasma essential AA levels after the meal replacement and AA drink for time point 180–420 min.

Plasma arginine concentration increased to a greater extent following consumption of the AA drink than the meal replacement drink at time points from 200 to 340 (all *P*‐values ≤ 0.016; Fig. 2). Similarly, plasma lysine concentrations were higher following the AA drink as opposed to meal replacement drink at all time points from 200 to 360 (all *P*‐values ≤ 0.046; Fig. 3). Plasma essential amino acid concentrations increased significantly after consumption of the AA drink compared with the meal replacement drink at time points 200–420 (all *P*‐values ≤ 0.005; Fig. 4). Branched‐chain (leucine, valine, and isoleucine) amino acid concentrations were significantly increased following consumption of the AA drink. Furthermore, a small but significant increase in plasma methionine and phenylalanine concentrations was found for the AA drink (all *P*‐values ≤ 0.37, and *P* ≤ 0.046, respectively). In contrast, plasma histidine concentrations increased to a greater extent following consumption of the meal replacement drink (all *P*‐values ≤ 0.039).

## Discussion

In this randomized intervention study, ingestion of the meal replacement drink significantly increased plasma HGH concentration in elderly women with heart failure, while an amino acid drink designed to stimulate an anabolic response in skeletal muscle did not result in an increase in plasma HGH. Plasma arginine and lysine concentrations, the two amino acids thought to be the most potent stimulators of HGH release (Chromiak and Antonio 2002), were significantly higher after consumption of the AA drink compared to the meal replacement drink.

HGH is secreted from the pituitary gland in a pulsatile manner and exerts its effect either directly or indirectly on the heart by stimulating the production of the somatomedin insulin‐like growth factor‐1 (IGF‐1), which promotes anabolic metabolism (Anker et al. 2001; Cicoira et al. 2003; Colao 2008). Various factors including training status, sex, age, diet, amino acids consumed, dosages and the mode of consumption [oral vs. intravenous] could possibly influence the HGH response to amino acid and protein administration (Chromiak and Antonio 2002; Stout 2002). In this study, we studied a rather homogeneous population and therefore the differences in composition of the drinks is the most likely explanation of the differences observed in the plasma HGH responses, particularly because of the cross‐over experimental design we used.

### AA composition

The amount of dietary protein or the amino acid composition might influence the acute HGH response to protein consumption, since only selective amino acids have been shown to be effective HGH‐secretagogs when administered orally or intravenously (Chromiak and Antonio 2002). The branched‐chain amino acids leucine and valine have been found in previous studies to induce rather small (~10%) increases in HGH levels, whereas isoleucine did not affect the HGH release in adults (Knopf et al. 1965). In contrast, infusion of the amino acids arginine, methionine, phenylalanine, lysine, and histidine stimulated relatively large increases (~8 to 22‐fold) in circulating HGH levels (Knopf et al. 1965; Tanaka et al. 1991; Chromiak and Antonio 2002; Collier et al. 2005). For this reason, we compared the ability of a protein‐based drink and an amino acid based drink on stimulating HGH secretion and their effect on plasma amino acid concentrations. The AA drink induced significantly higher increases in plasma arginine, lysine, methionine, and phenylalanine concentrations as compared to the meal replacement drink. However, histidine concentration was increase more following the meal replacement.

The failure of the AA drink to stimulate HGH release is in contrast to the results from previous studies showing that the amino acids arginine, lysine, methionine, phenylalanine, and histidine induce large increase in HGH levels (Tanaka et al. 1991; Chromiak and Antonio 2002; Collier et al. 2005). One possible explanation is that certain AAs inhibit the action of AAs that are positive HGH secretagogs. Another possibility is that other components of the meal replacement, that is, vitamin A, riboflavin, biotin, phylloquinone, cyanocobalamin, and vitamin D3 added to the meal replacement might have induced the HGH response. Studies investigating the effect of these components on HGH production are scarce. However, the results of a few studies suggest the possibility that biotin (Baez‐Saldana et al. 2009), phylloquinone (Kanellakis et al. 2012), and vitamin D3 (Ameri et al. 2013; Isgaard 2016) positively affect HGH production. It is also possible that the advanced age of our subjects, coupled with the physiological stress of heart failure, decreased the normal responsiveness to stimulation of HGH release by amino acids.

In addition to the protein and vitamins contained in the meal replacement drink, other macronutrients might have influenced the HGH response. Previous studies reported that the intake of protein, carbohydrates and fat all independently influence the secretion of HGH (Volek 2004). Studies reported that carbohydrate‐rich meals, which increases blood glucose levels, generally decrease blood HGH levels. This decrease in HGH level might be followed by a rebound hypoglycemia‐induced rise in HGH levels (Blackard et al. 1971; Holt et al. 2003; Volek 2004). However, rebound hypoglycemia was not a factor in our experiment. Moreover, it has been shown that increased plasma fatty acids levels inhibit HGH secretion (Fineberg et al. 1972; Volek 2004). Thus, we assume that the carbohydrates and fat in the meal replacement drink did not cause the significant increase in HGH secretion after consumption of the drink.

One limitation of this study is the absence of plasma IGF‐1 concentration analyses. Although the meal replacement significantly increased average HGH levels in these elderly women with heart failure. However HGH resistance is a common feature in patients with (severe) heart failure and therefore we are unable to conclude that significant increases in plasma HGH levels resulted in a beneficial impact on the heart (Volterrani et al. 2000). On the other hand, HGH resistance is commonly accompanied by higher baseline HGH levels and low IGF‐1 levels, and we observed 12‐fold higher baseline HGH values for one subject (1127 & 2370 pg/mL compared to an average of 90 and 158 pg/mL, respectively) (Jenkins and Ross 1998; Volterrani et al. 2000; Anker et al. 2001; Cicoira et al. 2003). For this reason, we assumed that seven out of eight participants did not yet develop HGH resistance and that the increase in HGH secretion might beneficially affect these patients.

Comparison of an AA‐based drink with a complete meal substitute drink could be criticized for the discrepant composition of the two beverages. The design of the study was anticipated to result in a stimulation of HGH by the AA drink, but not by the meal replacement. Such a result would support the role of the change in amino acid concentrations, since only the AA formulation increased the plasma amino acid concentrations. However, since the results were contrary to expectation, it is difficult to conclude what aspect of the meal replacement was responsible for the increase in HGH. However, this issue does not diminish the significance of the results, as we were primarily interested in the comparison of the postresponse versus the pre value with both drinks, rather than the between‐drink comparison. The most important observation of this study was that a commonly available meal replacement beverage was shown to effectively stimulate HGH secretion in a vulnerable group of elderly women. Is it unclear why the AA formulation failed to a response, but suggests a resistance to the normal stimulatory effect of amino acids. This would be consistent with the low basal levels HGH in these subjects, and imply that it was other components in the meal replacement that were the secretagogs. In any case, our results suggest that the anabolic effect of amino acids in elderly individuals with heart failure is not due to a concomitant increase in HGH concentration.

## Conclusion

Consumption of a meal‐replacement beverage significantly increased HGH concentration in elderly women with heart failure. This increase in HGH concentration is apparently not due to increases in plasma arginine and lysine concentrations, but caused by vitamins or other nutrients included in the meal replacement.

## Conflict of Interest

There are no conflicts of interest to declare by any of the authors of this manuscript.
